# Marsupials and monotremes possess a novel family of MHC class I genes that is lost from the eutherian lineage

**DOI:** 10.1186/s12864-015-1745-4

**Published:** 2015-07-22

**Authors:** Anthony T Papenfuss, Zhi-Ping Feng, Katina Krasnec, Janine E Deakin, Michelle L Baker, Robert D Miller

**Affiliations:** Bioinformatics Division, The Walter and Eliza Hall Institute of Medical Research, Parkville, VIC 3052 Australia; Department of Medical Biology, University of Melbourne, Melbourne, VIC 3010 Australia; Peter MacCallum Cancer Centre, East Melbourne, VIC 3002 Australia; Sir Peter MacCallum Department of Oncology, University of Melbourne, Melbourne, VIC 3010 Australia; Center for Evolutionary and Theoretical Immunology, Department of Biology, University of New Mexico, Albuquerque, NM 87131-0001 USA; Research School of Biology, Australian National University, Canberra, ACT 2601 Australia; Institute for Applied Ecology, University of Canberra, Canberra, ACT 2601 Australia; Australian Animal Health Laboratory, CSIRO, East Geelong, VIC 3219 Australia

## Abstract

**Background:**

Major histocompatibility complex (MHC) class I genes are found in the genomes of all jawed vertebrates. The evolution of this gene family is closely tied to the evolution of the vertebrate genome. Family members are frequently found in four paralogous regions, which were formed in two rounds of genome duplication in the early vertebrates, but in some species class Is have been subject to additional duplication or translocation, creating additional clusters. The gene family is traditionally grouped into two subtypes: classical MHC class I genes that are usually MHC-linked, highly polymorphic, expressed in a broad range of tissues and present endogenously-derived peptides to cytotoxic T-cells; and non-classical MHC class I genes generally have lower polymorphism, may have tissue-specific expression and have evolved to perform immune-related or non-immune functions. As immune genes can evolve rapidly and are subject to different selection pressure, we hypothesised that there may be divergent, as yet unannotated or uncharacterised class I genes.

**Results:**

Application of a novel method of sensitive genome searching of available vertebrate genome sequences revealed a new, extensive sub-family of divergent MHC class I genes, denoted as *UT*, which has not previously been characterized. These class I genes are found in both American and Australian marsupials, and in monotremes, at an evolutionary chromosomal breakpoint, but are not present in non-mammalian genomes and have been lost from the eutherian lineage. We show that *UT* family members are expressed in the thymus of the gray short-tailed opossum and in other immune tissues of several Australian marsupials. Structural homology modelling shows that the proteins encoded by this family are predicted to have an open, though short, antigen-binding groove.

**Conclusions:**

We have identified a novel sub-family of putatively non-classical MHC class I genes that are specific to marsupials and monotremes. This family was present in the ancestral mammal and is found in extant marsupials and monotremes, but has been lost from the eutherian lineage. The function of this family is as yet unknown, however, their predicted structure may be consistent with presentation of antigens to T-cells.

**Electronic supplementary material:**

The online version of this article (doi:10.1186/s12864-015-1745-4) contains supplementary material, which is available to authorized users.

## Background

The major histocompatibility complex (MHC) is a region unique to the genomes of jawed vertebrates and contains genes that are critical to the generation of immune responses. It is the most gene dense and polymorphic region in the genome (reviewed in [1]). The MHC is named for its role in recognition of ‘self’ and ‘non-self’, and was first identified in connection with tumour transplant rejection [2]. Genes in the MHC are also associated with resistance to infectious diseases, autoimmunity, reproductive success, inflammatory response and innate immunity (reviewed in [3, 4]).

The genes of the MHC are sub-divided into class I, II and III. The MHC class I genes are particularly noteworthy for having undergone gene duplication and divergence, resulting in an extended gene family whose members perform a broad range of functions. The classical role of class I molecules is to present endogenously-derived peptides to CD8^+^ T cells to stimulate cytotoxic responses against virus-infected or tumour cells. The class I molecules performing this role are sometimes referred to as classical MHC class I. Examples of classical class I genes include *HLA-A*, *-B* and *-C* in humans and *H2-K*, *H2-D* and *H2-L* in mouse. Classical MHC class I genes are generally broadly expressed in nucleated cells and highly polymorphic. Class I molecules performing other functions, collectively known as non-classical MHC class I, generally have low polymorphism, may have tissue-specific expression and in some cases have evolved functions other than antigen-presentation, including immuno-regulatory and non-immune roles. Examples of non-classical class I genes include *HLA-E*, *-F* and *-G* in human, *B1* and *Qa1* in mouse, as well as *MIC*. The function of non-classical molecules is not limited to the immune system. The *HFE* gene, for example, serves as part of the transferrin complex involved in iron storage (reviewed in [5]). Others, such as the neonatal Fc receptor, *FcRN*, that transports maternal IgG to fetal or neonatal mammals, has a role in the immune system that is distinctly different from conventional class I (reviewed in [6]). Typically, classical and some non-classical genes are located in the MHC, although many of the non-classical are located elsewhere in the genome [7].

In humans, the MHC is located on chromosome 6p [1]. Additionally, there are three regions of the genome that are paralogues of the MHC, indicative of the two rounds of whole genome duplication thought to have occurred in early vertebrate evolution [8]. These paralogous regions are located on chromosomes 1q, 9q, and 19p. They contain additional non-classical class I genes, including the *CD1* gene family, *MR1* and *FCGRT*. Other non-classical class I genes are found on chromosome 20 (*PROCR*), chromosome 7 (*AZGP1*) and chromosome 6q (*ULBP* and *RAET* families), suggesting that duplication and translocation have acted to further distribute MHC class I genes throughout the genome.

In other species, similar processes have acted to spread class I genes from the MHC. Two tightly linked, classical class I-like genes (*UB* and *UC*) in the opossum, *Monodelphis domestica,* for example, were translocated outside the MHC although they remain syntenic to the MHC on chromosome 2 [9, 10]. In a more extreme example, in the tammar wallaby, *Macropus eugenii,* the classical class I-like genes have been completely translocated out of the MHC and are distributed across multiple chromosomes [11].

**Fig. 1:**
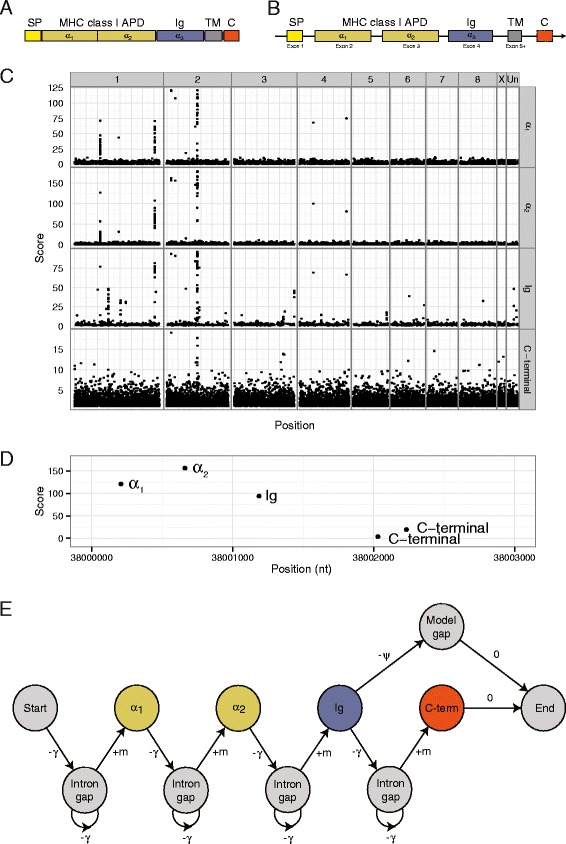
Sensitive pan-genome search for MHC class I genes. **a** The canonical domain structure of MHC class I proteins and (**b**) genes. **c** The location in the opossum genome and score of matches to profile hidden Markov models representing the antigen-presenting domain (split into α_1_ and α_2_ regions), C-type immunoglobulin domain and C-terminal domain. **d** Example of a high-scoring run of α_1,_ α_2_, Ig and C-terminal domains in the opossum genome. **e** Finite state automata of the alignment algorithm to search for runs of α_1,_ α_2_, Ig and C-terminal domains, taking domain score and distance between domains into account. The nodes (circles) show match states. Symbols on edges show scores/penalties: +m is the match score, which is based on the HMM match score; -γ is a distance-dependent affine gap penalty, which models introns and allows the alignment to skip over matches that interrupt a run of domains; -ψ is a constant penalty for dropping the C-terminal domain

Both classical and non-classical class I molecules have a conserved and distinctive protein domain structure. MHC class I genes typically have 5–9 exons encoding proteins with well-defined domain organization (Fig. 1a and 1b). The first exon encodes a signal peptide. Exons 2 and 3 encode the α_1_ and α_2_ domains, which together make up the antigen-presenting domain (APD). An immunoglobulin domain (Ig or α_3_) is encoded by exon 4. Additional exons may encode one or more transmembrane domains and the final exon contains a conserved cytoplasmic domain at the C-terminal of some MHC class I genes. The α_1_, α_2_ and Ig domains are the hallmark of MHC class I genes. However, different isoforms of some MHC class I genes exist. These may splice out some of these domains to produce other membrane bound versions of the protein or secreted forms. Additionally, the UL16-binding protein (*ULBP*) and retinoic acid early transcript (*RAET*) families, known in eutherians, are MHC class I-related genes that lack immunoglobulin domains and may utilize a GPI-anchor, rather than a transmembrane domain [12–15].

To better understand the evolution of MHC class I genes, particularly in mammals, we undertook to catalogue the class I genes. Here, we describe a sensitive comparative genomics analysis of MHC class I genes spanning vertebrate life. This was achieved using a novel approach based upon combining profile hidden Markov models (HMMs), which represent the separate domains characteristic of MHC class I genes. Our results reveal a new sub-family of MHC class I genes in marsupials and monotremes, which are not found in non-mammals and have been lost from the eutherian lineage. We show that these genes are transcribed in immune tissues in the gray short-tailed opossum, tammar wallaby, brushtail possum and Tasmanian devil. Structural homology mapping is used to begin to investigate the function of these genes.

## Methods

### Collection of annotated protein sequences

Predicted MHC class I proteins were identified and extracted from the Ensembl genebuilds (Release 75) of a selection of species spanning the gnathostomes, a jawless vertebrate and 3 invertebrate species. Protein sequences from human [16], mouse [17], dog [18], cow [19], opossum [20], wallaby [21], Tasmanian devil [22], platypus [23], chicken [24], zebra finch [25], turkey (The Turkey Genome Consortium), green anole lizard [26], *Xenopus tropicalis* [27], zebrafish [28], pufferfish [29], lamprey [30], sea squirt [27], fruitfly [31] and yeast [32] were searched using profile HMMs representing the MHC class I APD (PFAM:PF00129 and SUPFAM:0045513), C1-type Ig domain (PFAM:PF07654) and MHC class II β domain (PFAM:PF00969) using HMMer version 2 (fs and ls) and HMMer version 3 (PFAM models only). The separate domain searches were integrated and MHC class I proteins predicted using a simple heuristic: proteins were annotated as predicted MHC class I proteins if they had a significant match to the MHC class I APD (E-value < 10^-5^) or a weak match to the APD (score > 0) and a significant match to the Ig domain model (E-value < 10^-5^) in the correct order, with the additional requirement that the MHC class I APD model score is higher than the MHC class II β domain model score. Where a gene had multiple isoforms, the longest protein was selected as representative. The most sensitive approach (based on the number of proteins matched) used the SUPFAM MHC class I APD model, the PFAM Ig domain and HMMer2 in fs-mode. ULBPs and RAETs, which may not possess immunoglobulin domains, were identified by searching with the MHC class I APD HMM only.

### Sensitive genome search

Predicted MHC class I proteins were used to construct more sensitive custom profile HMM models in HMMer2. The 6-frame translations of the human (hg19), mouse (mm9), dog, cow, opossum (mondom5), wallaby (Meug_1.0), Tasmanian devil (assembly 7), platypus (OANA5), chicken, zebra finch, turkey, green anole, *Xenopus tropicalis*, zebrafish, tetraodon, lamprey, sea squirt, fruitfly and yeast genome sequences (Ensembl Release 75) were searched using profile HMMs representing the MHC class I APD (PFAM:PF00129, SUPFAM:0045513 and a custom model), C1-type Ig (PFAM:PF07654 and the custom model), C-terminal (PFAM:PF06623) and MHC class II β (PFAM:PF00969) domains with HMMer (version 2) with an E-value threshold of 10. Local alignment models (fs) were used.

The coordinates of predicted domains in the 6-frame translation were then transformed back to genomic coordinates. Genomic regions matching the first half of the MHC class I domain model were annotated as α_1_ domains, while features matching the second half were annotated as α_2_ domains. Regions also matching MHC class II β domains were removed if the class II match scores were greater than the class I match scores.

Genomic regions containing matches to the α_1_, α_2_, Ig and C-terminal domains with the correct orientation and order and intron-like separation were identified by aligning a model representing the canonical domain architecture of class I genes to the predicted domains (Fig. 1). The alignment algorithm was implemented using dynamic programming on the sequence of symbols, α_1_, α_2_, Ig and C-terminal, of predicted domains and taking into account their scores and the gaps between them. It used weighted HMMer scores as match scores. The weights were selected to approximately normalise the contributions from each domain (weights were α_1_: 1, α_2_: 1, Ig: 2, C-terminal: 20). An affine gap penalty was used to model introns with gaps shorter than 5000 nt penalty-free and calibrated so that a 20,000 nt gap gets a penalty of 300. Mismatches are effectively disallowed by applying a very large mismatch score (−20,000), but the affine gap function can skip over mismatching domains. Parameters were selected to have maximum sensitivity on the well-annotated human MHC class I genes and then tested on the mouse (positive control) and lamprey and sea squirt genomes (negative controls).

The method is summarized in Additional file 1: Figure S1 and code is available at https://github.com/papenfuss/MHC-clogs.

### Phylogenetic analysis

Multiple sequence alignments of predicted peptide sequences were generated using Clustal Omega [33, 34] and edited in jalview [35].

The phylogeny of the 449 predicted MHC class I genes identified in the representative jawed vertebrates was inferred using the Jones, Taylor and Thornton (JTT) model [36] in BEAST2 [37]. A discrete Gamma distribution with 4 categories was used to model evolutionary rate differences among sites. Four Markov chains were run for 3,000,000 steps each starting from random trees. Trees were output every 1000 steps. The consensus tree was estimated from the last 500,000 steps of the 4 chains.

To infer the evolutionary history of 30 selected human, mouse, marsupial and monotreme MHC class I genes, and the gene tree of the 46 *UT* family members, the best phylogenetic model was first selected using PROTTEST3 [38]. In both cases, the best model based on Akaike Information Criterion (AIC) was the JTT method [36] with invariant sites, gamma rate distribution, and empirical amino acid frequency (JTT + IGF). Phylogenetic trees were estimated using the maximum likelihood method with MEGA5 [38]. The bootstrap consensus tree inferred from 500 replicates was taken to represent the evolutionary history of the genes analysed. A discrete Gamma distribution was used to model evolutionary rate differences among sites with 4 categories. The rate variation model allowed for some sites to be evolutionarily invariable. The *UT* gene tree and the species tree were reconciled using NOTUNG [39] to identify gene duplication and loss events.

### BAC library screening

Overgo probes representing each of the wallaby and platypus novel class I genes were designed from genomic sequence using the Overgo Maker program. The specificity of the resulting overgo probes was judged by using the 40 bp probe sequence to BLASTN search the tammar wallaby or platypus genomes. All overgo probes used to screen the BAC libraries are listed in Additional file 1: Table S1. Overgo probes were radioactively labelled, pooled and hybridised to tammar wallaby (Me_KBa; Arizona Genomics Institute) BAC library filters as previously described [11]. Positive BACs from this initial screening were spotted onto Hybond N+ subjected to a further round of screening with individual probes as previously described [40].

### Fluorescence in situ hybridisation

DNA from each positive Bacterial Artificial Chromosome (BAC) clone was directly labelled with either SpectrumOrange or SpectrumGreen deoxyuridine triphosphate (dUTP; Abbott Molecular Inc., Des Plaines, IL, USA). Labelled BACs were hybridised to male metaphase chromosomes spreads, visualised and imaged as previously described protocol described [40].

### RT-PCR of predicted transcripts in opossum

Coding sequences of *mdUT* genes were amplified by targeted PCR with primers (Additional file 1: Table S2) designed based on predicted exon two and three gene sequence, using a cDNA library constructed from opossum thymus mRNA. The PCR was done using Advantage HF 2 PCR kit (Clontech, Mountain View, CA), with the following parameters for all primers: 94 °C for 1 min, 35 cycles of 94 °C for 30 s and 61 to 65.1 °C gradient for 4 min, and 68 °C for 5 min. The amplified DNA was then ligated into the pCR4-TOPO TA vector, transformed into One Shot Chemically Competent TOP10 *E. coli*, and incubated with 250 μL LB medium at 37° while shaking for 1 h (Invitrogen, Carlsbad, CA). A total of 120 μL of the transformed cells were then plated on ampicillin agar plates and incubated between 12–18 h at 37 °C. A minimum of 8 clones per plate were chosen, and plasmid DNA were generated using the boiling lysis method. Both the forward and reverse strands were sequenced with BigDye Terminator v3.1 Cycle Sequencing Kit (Invitrogen, Carlsbad, CA) Analysis of the sequences was done using Sequencher 5.0 (Gene Codes, Ann Arbor, MI).

### Searching marsupial immune tissue transcriptome data

To find support for the expression of *UTs* in several marsupials, sequencing data from the following immune tissue cDNA or Expressed Sequence Tag (EST) libraries were searched: Roche 454 sequencing data from tammar wallaby, *Macropus eugenii*, thoracic and cervical thymus cDNA libraries [GenBank:SRX019250,SRX019249] [41]; Roche 454 sequencing data from Tasmanian devil, *Sarcophilus harisii*, spleen and lymph node cDNA libraries [EMBL:PRJEB7940]; Roche 454 sequencing data from the opossum, *Monodelphis domestica*, thymus cDNA libraries (Katina Krasnec and Robert Miller, unpublished data); 17,818 ESTs from brushtail possum, *Trichosurus vulpecula,* spleen, lymph node and stimulated splenocytes [GenBank:LIBEST_019237]; and a small set of 1319 ESTs from a northern brown bandicoot, *Isoodon macrourus,* thymus ESTs [GenBank:EE743888-EE745206] [42].

Reads from each library were aligned to predicted tammar wallaby *UTs*, or Tasmanian devil *UTs*, in the case of the devil spleen and lymph libraries, using BLASTN. An E-value threshold of 10^−5^ was used and only a single best hit was recorded.

### Structural homology modelling

Structure prediction used the I-TASSER method [43]. Structural similarity or divergence was evaluated by a pairwise root mean square deviation (RMSD) value upon superposition of the backbone Cα trace from the two groups of structurally equivalent atoms in MHC class I α_1_ and α_2_ domains. Structure visualization and the RMSD calculation are using Pymol (http://www.pymol.org/).

## Results

### Sensitive peptide searches for MHC class I proteins

We first set out to identify all annotated MHC class I proteins in 15 representative species sampled from across vertebrate life. The selected species comprised human, mouse, dog, cow, three species of marsupials with sequenced genomes, platypus, three avian species, a lizard, a frog, and two fish species. Additionally, we selected 4 eukaryotic species known to lack MHC class I genes as negative controls (lamprey, sea squirt, fruitfly and yeast). Predicted protein sequences from these species were obtained from Ensembl and searched using profile HMMs representing the MHC class I APD and the C1-type Ig domain, which are characteristic of MHC class I genes, and the MHC class II β domain, with HMMer. The separate domain searches were integrated and MHC class I proteins predicted using a simple heuristic: proteins were annotated as predicted MHC class I proteins if they had a significant match to the MHC class I APD or a weak match to the APD and a significant match to the Ig domain model in the correct order, with the additional requirement that the MHC class I APD model matched with higher score than the class II β domain model. MHC class I genes frequently encode multiple isoforms; in these cases, we selected the longest protein as the representative protein. A variety of HMMs were tested (e.g. PFAM, SUPFAM and iteratively constructed custom models; see Methods for details) and the most sensitive combination was adopted.

Our search identified 348 MHC class I proteins across the 15 jawed vertebrate species searched (summarized in Table 1). This included all 24 known human and 41 mouse MHC class I proteins with no false positives. Searches of several negative controls—lamprey, sea squirt*,* fruitfly and yeast*—*did not identify any MHC class I proteins. Aligning all PFAM-A domain models to the set of predicted MHC class I proteins using hmmpfam showed that for each protein the strongest matches consisted only of the MHC class I APD, Ig and in some cases the conserved MHC C-terminal domains, with no other unexpected high quality matches. MHC class II genes were never misidentified as class I genes in the searches of any jawed vertebrate protein databases. Taken together these observations indicate the approach has high sensitivity and specificity.

**Table 1:** Summary of the number of MHC class I genes across species. The number MHC class I genes identified in each species by searching annotated proteins using customized

Species	Number of predicted MHC class I genes		
	Protein search	Genome search	Merged total
Human	24	26	33
Mouse	41	49	55
Dog	19	21	24
Cow	47	39	55
Opossum	28	40	47
Tammar wallaby	17	35	41
Tasmanian devil	22	23	25
Platypus	19	10	21
Chicken	24	21	26
Zebrafinch	11	3	11
Turkey	7	3	7
Green anole	25	19	26
Frog	26	31	32
Zebrafish	28	31	33
Tetraodon	10	10	13
Lamprey	0	0	0
Sea squirt	0	0	0
Fruitfly	0	0	0
Yeast	0	0	0
	348	361	449

### Sensitive genome searches for MHC class I genes

Next, we set out to identify any unannotated MHC class I genes in these genomes using a highly sensitive search method designed to take advantage of the conserved exon/domain organisation of MHC class I genes (Fig. 1a). Profile HMMs representing the MHC class I APD, C1-type Ig, MHC C-terminal, and MHC class II β domains were used to search the six-frame translation of each genome. The domain matches in the 6-frame translation were transformed back to genomic coordinates and the α_1_, α_2_, Ig and C-terminal domains within the model matches were identified. In each species, we found thousands of matches to these domains (summarized in Additional file 1: Table S3). For example in the opossum genome, we found 2127 matches to the α_1_ domain, 3571 matches to the α_2_ domain, 5028 matches to the Ig domain and 5546 matches to the MHC C-terminal domain. The majority of these matches had low scores. However, both isolated and clustered high scoring matches were also apparent (Fig. 1b). Genomic features matching the expected structure of an MHC class I gene, that is a chain of α_1,_ α_2_ and Ig domains and optionally a C-terminal domain on the same strand and at intron-like distances (for example Fig. 1c) were identified by aligning a canonical model of an MHC class I gene, taking match score and the gaps between domains into account (Fig. 1d, and Materials and methods for details). Once again, a variety of HMMs were tested (e.g. PFAM, SUPFAM, and custom models based on the protein search results; see Methods for details). The custom models were adopted as the most sensitive.

From the 388,409 domain matches across all species, the genome search identified 361 genomic features possessing the MHC class I gene structure (summarized in Table 1; Additional file 2: Table S4 for details). These included 26 putative MHC class I genes in the human genome, 49 in mouse, and 40 in the opossum. Again, searches of the negative controls identified no MHC class I genes, as one would expect. These genomic features included annotated genes, and both annotated and unannotated pseudogenes. Merging the protein and genome searches produced a total of 449 MHC class I genes and proteins across the species searched (Additional file 3: Table S5), including a total of 33 in human, 55 in mouse and 47 in the opossum.

The most dramatic differences between the results of searching annotated class I proteins and an unbiased search of the whole genome arose in the marsupials and monotremes. The annotation of the opossum genome (Ensembl Release 75) contains 28 MHC class I genes, but 40 putative MHC class I genes (genomic features with structural similarity to MHC class I genes) were identified in the sensitive genome search results. Seven of the annotated proteins were missed in the genome search, as the corresponding loci lack Ig domains. Fifteen of the loci identified by the genome search were unannotated in the Ensembl genebuild. In some cases, de novo gene predictions from genscan or evidence-based prediction with N-scan (UCSC Genome Browser, accessed 17 April 2015) did identify overlapping open reading frames, however, these annotations were typically of poor quality (data not shown) with multiple run-on annotations linking two or more MHC class I gene features. Five of these unannotated features contained in-frame stops, including opossum *CD1*, *UH*, and a *MIC*-like gene (*MIC2*). These in-frame stops may be due to sequencing errors in the draft opossum genome, polymorphisms in the individual sequenced or the fact that our model does not take splice sites into account and may erroneously include short segments of intronic sequence in the domain matches, resulting in the genomic feature going out of frame. In fact, CD1 is known to be a pseudogene in opossum [44] and does not show evidence of transcription; MIC2 also shows no evidence for transcription; while UH does show evidence of transcription (data not shown). Consequently, we retain all genes in our analyses. Thus, a total of 47 putative MHC class I genes were identified. A similar pattern emerged in other marsupial and monotreme genomes.

### Phylogenetic analysis

**Fig. 2:**
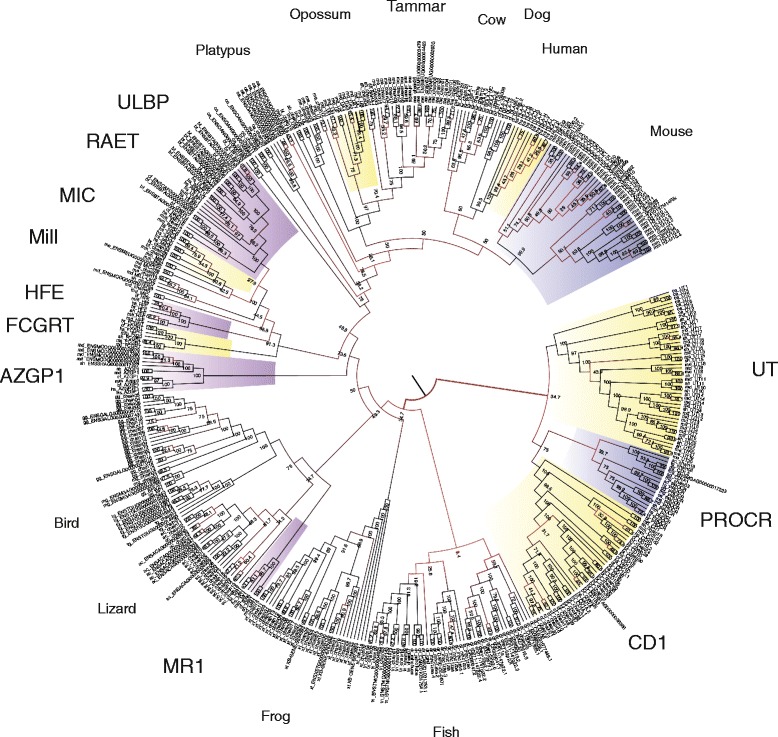
Phylogeny of class Is predicted in representative species spanning the jawed vertebrates estimated by MCMC on the JTT + IG model. Numbers at nodes represent the frequency with which that split is observed. Gene families are labelled around the outside. The species label shows the location of classical MHC class I for each species or group of species. Key gene families or species’ classical class I genes are highlighted in colour

To annotate these genes and understand the evolutionary relationship between them, we inferred the phylogenetic relationships between all MHC class I genes identified in the selected vertebrates using a Markov Chain Monte Carlo (MCMC) method on the JTT + IGF model. Four MCMCs were run (see Additional file 1: Figure S2 for traces of posterior probability) and the consensus tree from the last 500 steps of each run was taken to represent the evolutionary history of the genes (Fig. 2a). Additionally, a smaller phylogeny consisting of just human and opossum class I genes and the mouse Mill genes was also inferred by maximum likelihood (Additional file 1: Figure S3).

While support in parts of the trees is low, the phylogenies provide a number of insights into the evolution of MHC class I genes in vertebrates. The large tree provides additional evidence for the previous observation that the non-classical MHC class I gene family *MR1* is found only eutherians and marsupials [45]. Similarly, it suggests that the *FCGRT*, *HFE* and *AZGP1* gene families are specific to eutherians and marsupials. It demonstrates that the *PROCR* gene family is found across the amniotes. It suggests that MIC is duplicated in opossum (md_chain40), though this contains in-frame stops. The small tree supports the previous observation that marsupials may have a member of the *ULBP* gene family (ENSMODG00000015798) [46]. It identifies a possible expansion of *AZGP1* in opossum (ENSMODG00000024063, ENSMODG00000027380, ENSMODG00000028158, and ENSMODG00000029679). The phylogenies also reveals two new opossum MHC class I genes that are located in the MHC, but have not previously been identified, which we have denoted *UA3* and *UA4*. These appear to be closely related to *UA1* and *UA2*.

Strikingly, the phylogenetic tree identifies an extensive and entirely novel clade of MHC class I genes in marsupials and monotremes, which we have named *UT*. There are 17 *UT* family genes identified in the opossum genome, 9 in tammar wallaby, 13 in the Tasmanian devil and 7 in the platypus. The numbering of *UT*s is based on location in the gene cluster in the opossum and clear orthology, or lack of it in other marsupials. Platypus UTs are numbered independently as these appear to form a distinct clade. This is highlighted by the UT gene tree (Additional file 1: Figure S4), which was estimated using maximum likelihood with the JTT + IGF model and reconciled with the species tree using NOTUNG. No *UT*s were identified outside of the marsupials and monotremes in our searches.

### Chromosomal location

**Fig. 3:**
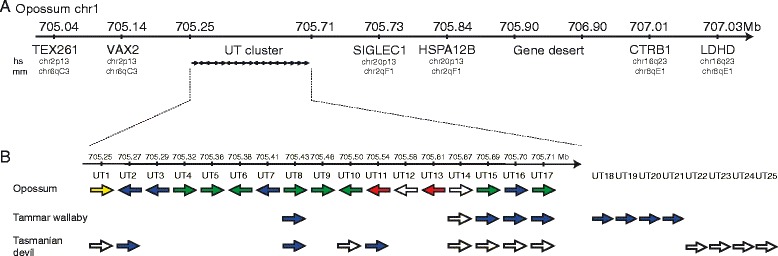
Comparative map of the *UT* gene cluster. **a** Genomic region containing the *UT* cluster in opossum showing the non-synteny of flanking genes between the opossum and human (hs) and mouse (mm) genomes. **b** Comparative map of *UT* cluster in opossum, tammar wallaby and Tasmanian devil. The fill colour summarises the evidence for expression: RT-PCR in opossum thymus detected (green) and not detected (red)*;* 454 sequencing data (blue); in-frame stop (yellow); not tested (white)

**Fig. 4:**
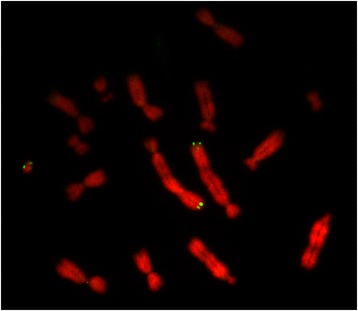
Fluorescence In-Situ Hybridisation showing location of *UT* cluster on chr1 in the tammar wallaby. A signal is also observed on the Y chromosome

The *UT* family of MHC class I genes is encoded in a gene cluster on chromosome 1 in the opossum genome (Fig. 3). This region is approximately 460 kilobases in size. Interestingly, the cluster is located at an evolutionary breakpoint and is flanked by genomic regions that share synteny with different chromosomes in human (chr2 and chr20) and mouse (chr6 and chr2). The tammar wallaby genome assembly (Meug1.0) is highly fragmented and scaffolds are not mapped to chromosomes. Fluorescence In-Situ Hybridization (FISH) shows that the *UT* gene cluster is also located on chromosome 1 in the tammar wallaby genome (Fig. 4), as predicted by conserved synteny between the tammar and opossum [47]. Interestingly, the FISH also shows a signal on the tammar Y chromosome. As all marsupial genomes sequenced were female, this locus was not detected in genome-wide searches and the significance of this signal is not yet understood. Based on the digital karyotype of the Tasmanian devil [22], the *UT* gene family is also located on chromosome 1.

### Sequencing and gene expression

Of the 17 putative opossum *UT* genes, the expression of 8 genes, consisting of *UT4, UT5, UT6, UT8, UT9, UT10, UT15,* and *UT17,* was confirmed in opossum thymus using RT-PCR (Additional file 1: Figure S5). Predicted sequences obtained from our sensitive search method were confirmed using RT-PCR to obtain amplicon sequences from within exons 2 and 3 (Additional file 4: Table S6). A further 4 *UT* loci, *UT2, UT3, UT7,* and *UT16*, were confirmed as expressed in Roche 454 sequencing data from an opossum thymus cDNA library (data not shown).

Transcription of tammar wallaby *UT8, UT15, UT16, UT17, UT18, UT19, UT20,* and *UT21* was confirmed in 454 data from thoracic and cervical tammar thymus cDNA libraries. There was support for Tasmanian devil *UT2*, *UT8,* and *UT11* in 454 cDNA data from devil spleen, but no UTs were detected in a lymph node library.

Limited transcriptome sequence data is available from immune tissues of other species of marsupial. An EST (id: 161106CS44009845FFFFF) from brushtail possum immune tissues with homology to *meUT2* was also identified, providing support for the existence of functional *UTs* in the possum. No *UT* transcripts were detected in BLASTN searches of 1318 bandicoot thymus EST library, probably due to the small size of this library. No platypus immune tissues transcriptome data was available.

### Homology mapping

**Fig. 5:**
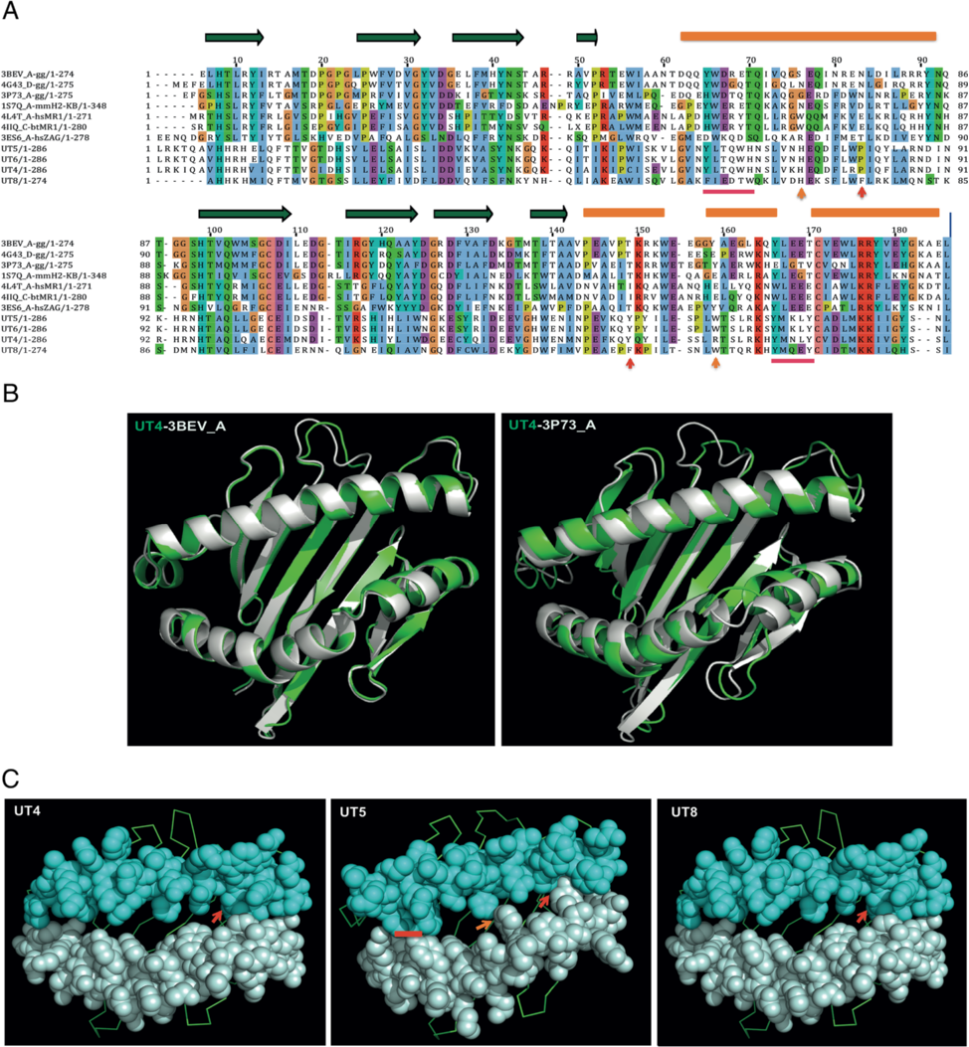
Predicted structure of UT proteins. **a** Sequence alignment of the α_1_ and α_2_ domains of *UT4, 5, 6, 8* with 7 of the top 10 structural analogs from Protein Data Bank (PDB) identified by I-TASSER. Orange bars show α-helices. Green arrows highlight β-strands. Major differences between the *UT*s and templates are indicated by arrows, or red lines, and the consequence of these on the protein structures are shown in Fig. 5c. **b** Overlay of the backbone of the peptide binding groove of *mdUT4* with its top 2 structural analogs, 3BEV_A (*ggB21*) and 3P73_A (*ggYF1*). **c** Superposed model structures of *mdUT4*, *5* and *8* with the *B21* template shows the antigen-binding groove is open, but possibly short. Filled sphere view shows the α-helices and ribbons show the β-sheets of the peptide-binding platform on the modeled protein structures. The residues indicated with arrows in Fig. 5a cause the binding grooves to be short or narrow (e.g. *UT4*: the distance between Pro81 Cγ to Tyr144 OH is 3.0 Å; *UT5:* the distance between Pro81 Cβ to Tyr144 OH is 2.9 Å, and a close hydrophobic contact between _64_ **LT**Q**W**_**67**_ and _161_ **M**N**LY**_**154**_; *UT8*: the distance between Phe75 Cε to Phe138 Cξ is 2.9 Å)

To investigate the function of *UT* family members we predicted the protein structure of selected *UTs* (opossum *UT4, UT5* and *UT8*) using homology modelling with the I-TASSER method [43]. Protein structures from the Protein Data Bank (PDB) that were closest to the predicted models comprised both classical and non-classical MHC class I genes from chicken, cow, mouse and human (Additional file 1: Table S7; Fig. 5a for an annotated sequence alignment of 7 of the top matches). The structure of the classical chicken MHC class I protein B21 (3BEV [48]) was the best match for *UT8* and appeared in the top 5 templates for all *UT*s examined. The backbone structural alignment of *UT4* with 3BEV and 3P73 [49], the top 2 structural analogs for *UT4* and *UT8*, are shown in Fig. 5b. The peptide-binding grooves of *UT4, 5* and *8* are shown in Fig. 5c.

## Discussion

MHC class I molecules have historically been defined by their function. The classical MHC class I typically presents peptide fragments derived from antigens to CD8^+^ cytotoxic T lymphocytes. This particular function is ubiquitous across the jawed vertebrates and is likely the primordial function of the class I protein. However, it is clear that members of the MHC class I family have evolved to perform other functions, often in lineage specific ways. Therefore, a more appropriate definition of an MHC class I molecule is its unique structure which is a heterodimer of a α-chain paired with β2-microglobulin. The MHC class I α-chain is composed of three extracellular domains. The α_3_ domain is an immunoglobulin domain, a protein fold that predates the origin of jawed vertebrates in evolution. The origins of the α_1_ and α_2_ domains that make up the antigen-binding groove are more enigmatic and appear unique to the MHC molecules. Searching the genomes of jawless vertebrates and invertebrates failed to uncover genes encoding α_1_- and α_2_-like domains, shedding no light on their evolutionary origin.

The diversity of functions that MHC class I molecules have evolved to perform demonstrate the plasticity of this protein structure. For example, FcRN, which functions as an IgG receptor in mammals, does not bind the Fc region using the antigen-binding groove. Rather that groove is fairly closed and the IgG binds to a combination of the outer face of the α_2_ and β2-microglobulin domains [50]. Such functional plasticity of a protein structure leads to the question of what other roles these molecules may have evolved to perform and how divergent they may have become. The results presented here demonstrate that highly divergent genes, based on nucleotide sequence can encode proteins that fold to produce the MHC class I α-chain structure. This is not to say that the *UT* genes are a product of convergent evolution. Indeed, the phylogenetic analyses places them squarely within the extended MHC class I family. The phylogenetic position of these genes, close to *CD1* and *PROCR*, suggests they are non-classical MHC class I genes, but diversity and expression also need to be assessed.

The presence of the *UT* family of MHC class I genes in both marsupials and monotremes is consistent with their being ancient and present in the most recent common ancestor of all living mammals. The common ancestor of marsupials and eutherians (placental mammals) lived approximately 165 million years ago [51]. It appears that after the divergence of these two lineages the *UT* family was lost in the eutherians, likely prior to the radiation of the modern eutherians. Without knowing the function of *UT* genes it is difficult to speculate on why they were dispensable in the eutherians. However this is not the first case where mammal-specific immune system genes have been lost in the eutherians. Both marsupials and monotremes have orthologues of a uniquely mammalian T cell receptor called TCRμ, which has been lost in the eutherian lineage [52, 53]. Given the classical role of MHC class I molecules interacting with TCR it is an intriguing possibility that there is a functional connection between the UT molecules and TCRμ, whereby UT present antigen to TCRμ^+^ T cells. Hence, the loss of one may have resulted in the loss of the other in eutherians. While possible this would not be consistent with current models of how TCRμ chains interact with antigen in an MHC independent manner [54]. Similar to *UT* loci, the *TCRμ* cluster is located in a region of the mammalian genomes that have a break in synteny between marsupials and eutherians [55]. It may be these gene families were independently lost due to being in regions of the genome subject to instability or rearrangement.

Other working hypotheses on the function of UT molecules are based on models predicting the structure of the region corresponding to an antigen-binding groove. Structurally, the UT proteins are most similar to the chicken B21 MHC class I molecule (3BEV), which binds peptide promiscuously [48]. The presence of some hydrophobic residues in the α-helices may make the effective binding size short or narrow. This may suggest the UTs present small peptide fragments, but is also consistent with a structure where the space between the α_1_ and α_2_ domains is relatively closed or alternatively, UT molecules may be involved in presenting hydrophobic antigens such as lipids. Marsupials have a homologue of the CD1 molecule that is normally involved in the presentation of glycolipids and lipoprotein antigens [56]. However, the marsupial gene is single copy and not orthologous to any of the known *CD1a, b, c, d,* or *e* genes found in eutherians [44]. Furthermore, in the opossum *M. domestica CD1* is a pseudogene [44]. It is possible that there has been less pressure to retain or diversify the *CD1* family in marsupials due to some functional overlap with the *UT* genes.

The region of chromosome 1 containing the *UT* cluster does not correspond to one of the four MHC paralogous regions. These paralogous regions are the remnant of the two rounds of whole genome duplication that occurred during vertebrate evolution [57]. In humans these regions are located on chromosomes 1, 6, 9, and 19, with chromosome 6 containing the *bona fide* MHC region. In the opossum, they are located on chromosomes 1, 2, and 3, with two paralogous regions on chromosome 2, one being the MHC proper [58]. The paralogous region on opossum chromosome 1 corresponds to human chromosome 9 and is syntenic to, but not identical to, the opossum *UT* region. Therefore, the extant *UT*s are the product of the novel expansion of diverging MHC class I genes in the marsupials and monotremes and likely originate from the duplication of an MHC class I gene in the ancestral mammal.

## Conclusions

Using a novel, boutique method to search the annotated proteins and genomes of a selection of species spanning vertebrate life for MHC class I genes with high sensitivity, we identified a new class I gene family, the *UT*s. *UT* family members are encoded in gene clusters on chromosome 1 of the opossum, tammar wallaby and Tasmanian devil genomes, and are present but have not been mapped in platypus. The region is located in a synteny break between marsupial and eutherian genomes. Homology modelling suggests *UT* genes have an open but short antigen-presenting groove, raising the possibility that they may present peptide epitopes or non-peptide fragments.

Further investigation of the expression and protein structure of UTs is needed to understand their function. This may be relevant to understanding the evolution of the vertebrate immune system, immune surveillance, and diseases affecting marsupials and monotremes, including Tasmanian devil facial tumour disease, chlamydia in koalas, and mucormycosis in platypus, which pose major threats to these species.

Finally, our boutique sensitive search method can be adapted to study other gene families and will also be of interest to comparative genomics researchers.

### Ethics statement

All procedures involving animals were approved by the Institutional Animal Care and Use Committee of the University of New Mexico and conducted under protocol number 07UNM005. No live surgeries were performed.

## Additional files

Additional file 1:
**Supplementary data (Figure S1-S5; Table S1-S3 & S7).** **Figure S1.** Sensitive protein and genome search method workflows. **Figure S2.** The posterior probability of the phylogeny of 449 predicted MHC class I peptides generated by 4 MCMCs using BEAST2, started from random trees and sampled every 1000 steps. **Figure S3.** Maximum likelihood tree (JTT + IGF model) of selected proteins including UT family members. Numbers at nodes indicate bootstrap support. **Figure S4.** UT gene family tree was estimated by maximum likelihood using the JTT + IGF model and reconciled with the species tree using NOTUNG. Predicted gene losses are shown in grey. Predicted duplications are indicated by a “D” at internal nodes. Bootstrap support is shown in red. **Figure S5.** Confirmation of expression by RT-PCR in the opossum thymus. **Table S1.** Overgo sequences used to isolate tammar wallaby and platypus BACs containing UT loci. **Table S2.** RT-PCR primers for opossum UT exons 2 and 3. **Table S3.** Number of domain matches found in the genomes of each species using custom profile hidden Markov models. **Table S7.** The pairwise backbone Root Mean Square Deviation (Å) between the α1 and α2 domains of the opossum UT4, UT5, and UT8 modeling structures, several of the top 10 closest structural analogs identified using I-TASSER, and selected classical and non-classical MHC class I proteins from human and mouse. Additional file 2: Table S4. Genome annotations of predicted MHC class I domains found by the sensitive genome search. Additional file 3: Table S5. Predicted MHC class I peptide sequences. Additional file 4: Table S6. Opossum *UT* exon 2 and 3 amplicon sequences.

## Abbreviations

AIC: Akaike Information Criterion
APD: Antigen-presenting domain
BAC: Bacterial Artificial Chromosome
cDNA: Complimentary DNA
EST: Expressed Sequence Tag
FISH: Fluorescence In-Situ Hybridization
GPI: Glycosylphosphatidylinositol
HMMs: hidden Markov models
Ig: Immunoglobulin
IgG: Immunoglobulin G
JTT: Jones, Taylor and Thornton model
JTT + IGF: JTT model with invariant sites, gamma rate distribution, and empirical amino acid frequency
MCMC: Markov Chain Monte Carlo
MHC: Major histocompatibility complex
PCR: Polymerase chain reaction
PDB: Protein Data Bank
RMSD: Pairwise root mean square deviation
