# Supplementary material for: Marsupials and monotremes possess a novel family of MHC class I genes that is lost from the eutherian lineage
Source: BMC Genomics. 2015 Jul 22;16(1):535. doi: 10.1186/s12864-015-1745-4 (PMC4509613; doi:10.1186/s12864-015-1745-4)
Supplement: Additional file 1: — Supplementary data (Figure S1-S5; Table S1-S3 & S7). Figure S1. Sensitive protein and genome search method workflows. Figure S2. The posterior probability of the phylogeny of 449 predicted MHC class I peptides generated by 4 MCMCs using BEAST2, started from random trees and sampled every 1000 steps. Figure S3. Maximum likelihood tree (JTT + IGF model) of selected proteins including UT family members. Numbers at nodes indicate bootstrap support. Figure S4. UT gene family tree was estimated by maximum likelihood using the JTT + IGF model and reconciled with the species tree using NOTUNG. Predicted gene losses are shown in grey. Predicted duplications are indicated by a “D” at internal nodes. Bootstrap support is shown in red. Figure S5. Confirmation of expression by RT-PCR in the opossum thymus. Table S1. Overgo sequences used to isolate tammar wallaby and platypus BACs containing UT loci. Table S2. RT-PCR primers for opossum UT exons 2 and 3. Table S3. Number of domain matches found in the genomes of each species using custom profile hidden Markov models. Table S7. The pairwise backbone Root Mean Square Deviation (Å) between the α1 and α2 domains of the opossum UT4, UT5, and UT8 modeling structures, several of the top 10 closest structural analogs identified using I-TASSER, and selected classical and non-classical MHC class I proteins from human and mouse. [file 12864_2015_1745_MOESM1_ESM.pdf]

Supplementary Data

Marsupials and monotremes possess a novel family of MHC class I genes that is lost from the eutherian lineage

Anthony T Papenfuss, Zhi-Ping Feng, Katina Krasnec, Janine E Deakin, Michelle L Baker, Robert D Miller

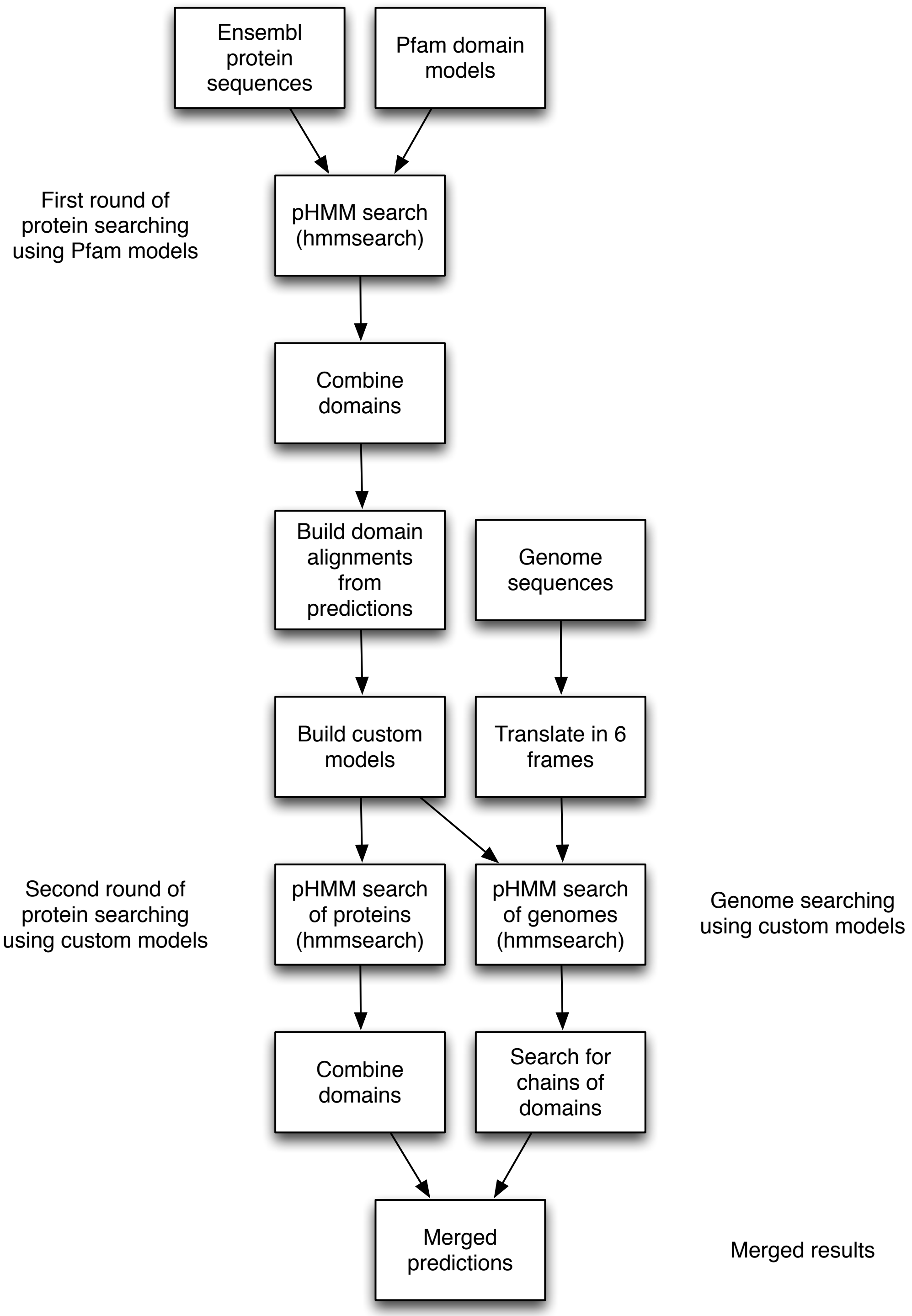

Figure S1. Sensitive protein and genome search method workflows.

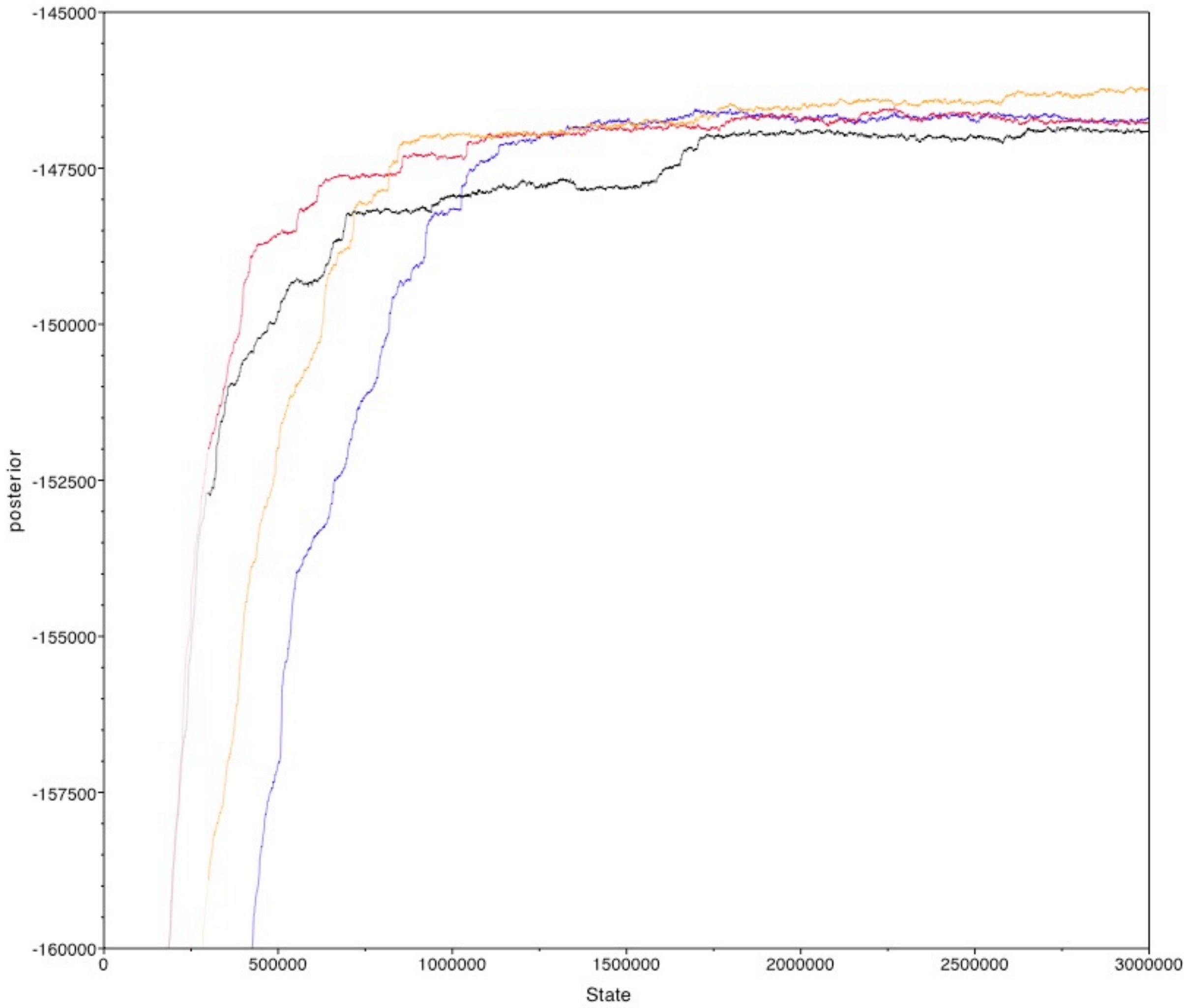

Figure S2. The posterior probability of the phylogeny of 449 predicted MHC class I peptides generated by 4 MCMCs using BEAST2, started from random trees and sampled every 1000 steps.

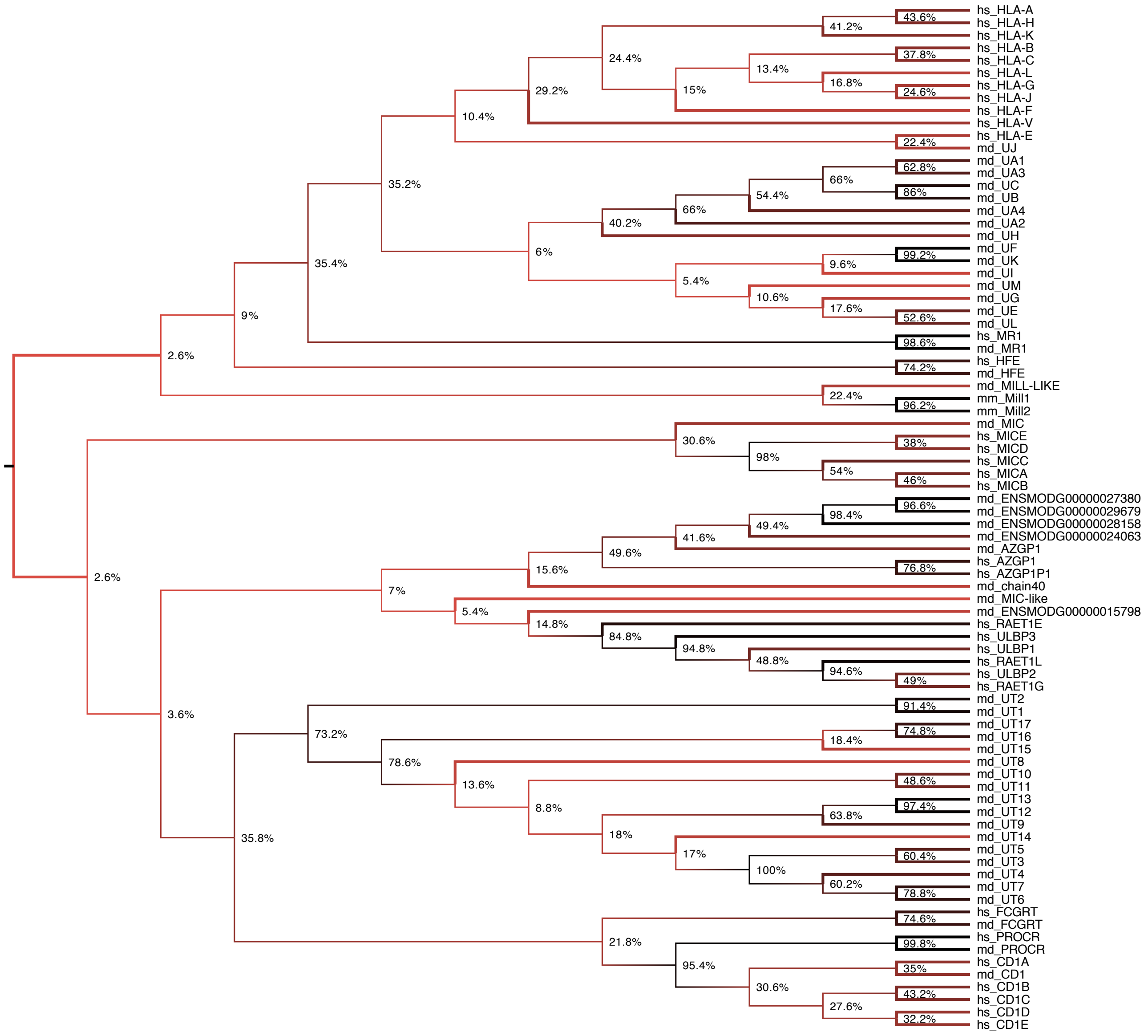

Figure S3. Maximum likelihood tree (JTT+IGF model) of selected proteins including *UT* family members. Numbers at nodes indicate bootstrap support.

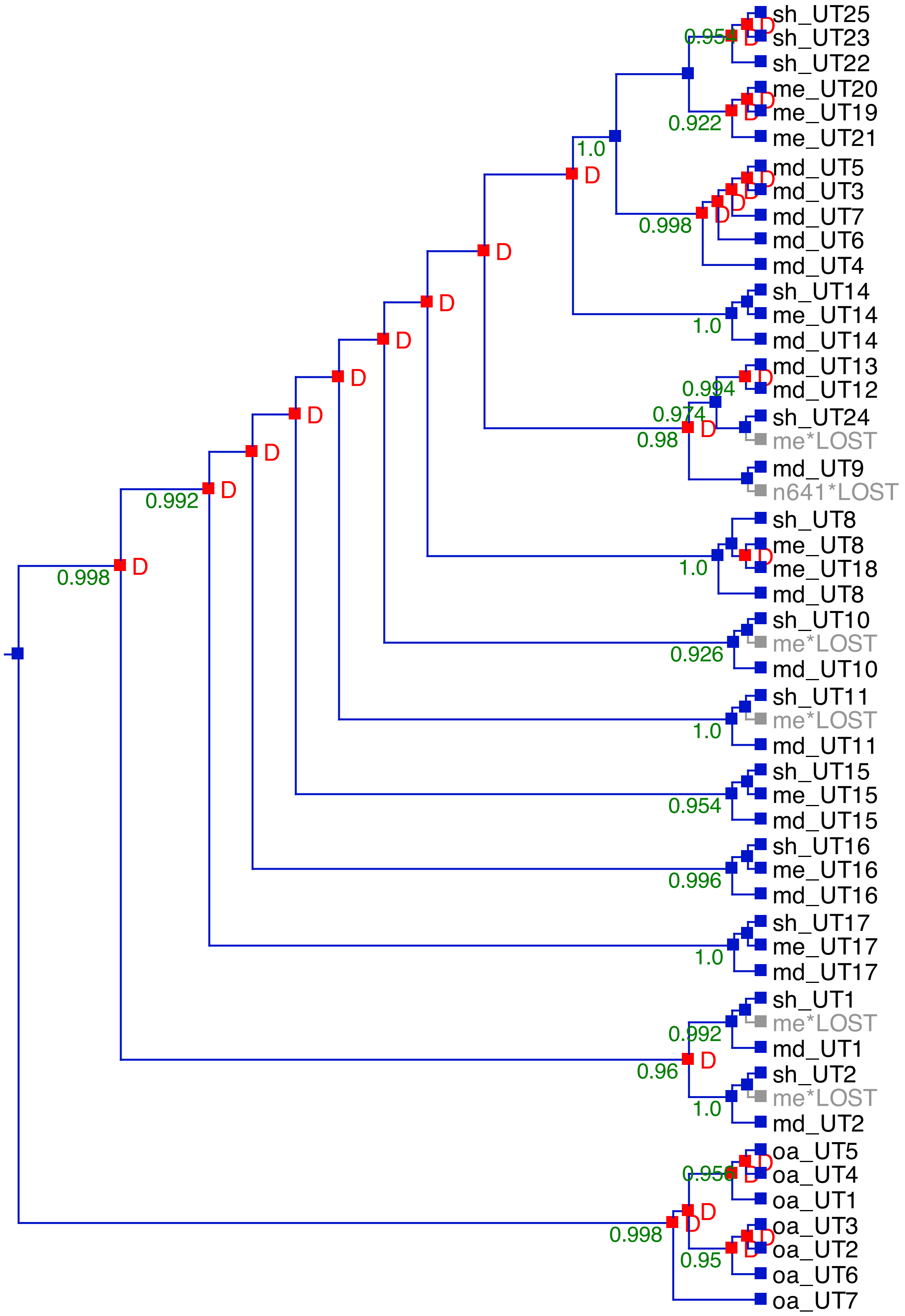

Figure S4. *UT* gene family tree was estimated by maximum likelihood using the JTT+IGF model and reconciled with the species tree using NOTUNG. Predicted gene losses are shown in grey. Predicted duplications are indicated by a “D” at internal nodes. Bootstrap support is shown in red.

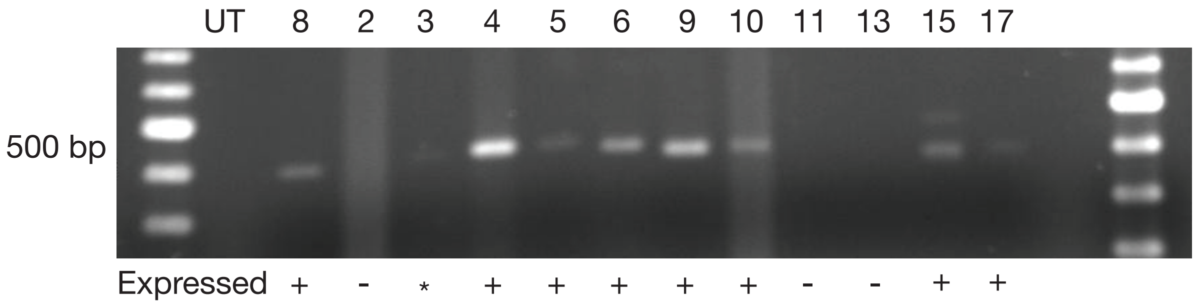

Figure S5. Confirmation of expression by RT-PCR in the opossum thymus.

Table S1. Overgo sequences used to isolate tammar wallaby and platypus BACs containing *UT* loci.

Gene symbol	Overgo A	Overgo B	BACs
me_UT21	CATGTGGGTCAGTTCACTGCAGTG	GAGAATGGATTGTGCCCACTGCAG	289L16, 370J4
me_UT20	CACCACAAGCACATAGCTCAGTTC	TTGTGCCCACTGCAGTGAACTGAG	289L16, 370J4
me_UT18	GTCTGAGGATCAGGATCAAAGAAC	GTCCTCTCCCTAGAATGTTCTTTG	No BACs
me_UT16	CACCCTTGAGATGTGTTTCACAGC	CTTTGTTGTACCTACTGCTGTGAA	334C11, 352G17, 432E9
me_UT19	GTTTGTGGGTCAGTTCACTGCAGT	TTTTGTGCTTGTGCCCACTGCAGT	289L16, 370J4
me_UT14	ATTTCACTGCAGTGGGCACAGCCA	AAGTTCAGCAGGGAGCTGGCTGTG	334C11, 352G17, 432E9
me_UT15	CAAGGTCTCTCAAACCTCATAGTC	CATCCACAGAACTGATGACTATGA	334C11, 352G17, 432E9
me_UT21	TAGGCCCATGAATGATGGATTTTC	CACTAGACAGCAGATAGAAAATCC	289L16, 370J4

Table S2. RT-PCR primers for opossum *UT* exons 2 and 3.

Gene	Exon 2/3 primers
mdUT1	5' GTTCTGCAGCTCACCACAAAC 3'<br>5' CATGGTGTCAATACAATATTCTTGC 3'
mdUT2	5' ACAGCCTGGAGATCCAGTTCA 3'<br>5' GTTCCAGGACAATCTTCAGAAA 3'
mdUT3	5' ATTCAGTCCACCACAGCCATA 3'<br>5' TTTTCCTCATCAAGTCAACACAA 3'
mdUT4	5' ACCACAGGCATGTGATTCAGT 3'<br>5' GCTTGAATATCCAACAATTTTCTTC 3'
mdUT5	5' ATTCAGTCCACCACAGGCATA 3'<br>5' TTATTTTCCTGATCCCGTCAAC 3'
mdUT6	5' ATTCAGTCCACCACAGACATGA 3'<br>5' TGATTTTCTTCATCAAGTCAGCA 3'
mdUT8	5' CATTTAGAATACCACTGGAATGAAG 3'<br>5' ACATCGACACAATATTGCTTAACG 3'
mdUT9	5' AGGCTTGAGGGTGAATTCATTG 3'<br>5' TGCAGGATTTTTCTTATCATTTCA 3'
mdUT10	5' ACAGATTACCACAGGCATGAAG 3'<br>5' TACTGCAGAATTTTCCTCATCA 3'
mdUT12	5' CAATCACAAGCATGAATTGC 3'<br>5' GAAGGATTTTCTGCATGACTCC 3'
mdUT14	5' CCACAGGCATGACTTCTATTTC 3'<br>5' CATGCCATCAATACAATCTTCC 3'
mdUT15	5' CCATCACAGCCATGAGATGTTT 3'<br>5' TCATTGGCTGGACACAATAGTC 3'

Table S3. Number of domain matches found in the genomes of each species using custom profile hidden Markov models.

**Species**	**Number of domain matches**			
	Class I APD	Ig	C-terminal	Class II beta
Human	8468	17738	25836	24437
Mouse	7718	19763	25160	24902
Cow	9752	17066	33669	22055
Dog	6663	18066	22088	21248
Opossum	2127	3571	5028	5546
Tammar wallaby	212	295	53	38
Tasmanian devil	3728	4147	4093	6992
Platypus	795	4651	5480	5469
Chicken	3075	8752	10813	11979
Zebrafinch	4069	10490	13807	14411
Turkey	2833	7236	9760	10959
Green anole	4528	8813	9732	14282
Frog	1541	4996	2673	8766
Zebrafish	4674	9489	10040	16523
Tetraodon	930	2887	4448	3706
Lamprey	263	927	658	328
Sea squirt	606	588	403	1537
Fruitfly	644	779	1455	1762
Yeast	108	107	118	178
	62734	140361	185314	195118

Table S7. The pairwise backbone Root Mean Square Deviation (Å) between the α_1 and α_2 domains of the opossum *UT4*, *UT5*, and *UT8* modeling structures, several of the top 10 closest structural analogs identified using I-TASSER, and selected classical and non-classical MHC class I proteins from human and mouse.

Protein		*UT4*	*UT5*	*UT8*	Comment
mdUT4	-	0	1.716	0.936	
mdUT5	-	1.716	0	1.449	
mdUT8	-	0.936	1.449	0	
ggYF1	3P73*	2	1.79	1.953	
ggB4	4G43*	1.02	1.368	1.22	
ggB21	3BEV*	1.041	1.969	0.501	Binds 11mer peptide
ggB21	2YF6*	3.356	1.884	0.871	Binds 10mer peptide
btMR1	4IIQ*	2.387	2.308	2.308	
mmH-2Kb1	1S7Q*	2.492	2.979	2.959	
hsMR1	4L4T*	3.399	3.402	3.296	
hsAZGP1	3ES6*	3.121	3.941	3.462	
hsFcRn	1EXU	1.941	2.881	4.288	
hsHFE	1A6Z	2.657	2.977	2.919	
hsMICA	1HYR	4.519	6.291	5.534	
hsMICB	1JE6	6.989	7.94	8.283	
hsULBP	1KCG	22.309	7.634	4.986	
hsCD1a	1ONQ	12.601	13.225	4.042	
hsCD1b	1GZQ	7.31	5.466	3.49	
hsPROCR	1L8J	6.17	6.669	4.545	

*From top 10 templates for all the UTs
